# Money, power, and mitochondria

**DOI:** 10.15252/embr.202255423

**Published:** 2022-05-25

**Authors:** Howy Jacobs

**Affiliations:** ^1^ Tampere University Tampere Finland; ^2^ La Trobe University Melbourne Australia

## Abstract

Can we devise ways for the ultra‐rich to put their wealth to good use?

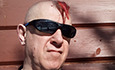

The world’s richest individuals and their wealth have recently come under the spotlight, with all those luxury yachts apparently owned by Russian oligarchs and their political friends veering to safe havens beyond the reach of sanctions; most of them vastly surpassing in value the cost of an advanced warship or the on‐off‐on saga of Elon Musk’s purchase of Twitter for a sum that exceeds the annual GDP of a mid‐sized country.

Even if their riches have been acquired through legal means and provide joy and sustenance to many, how can it be justified that a small number of individuals own such vast assets, when a substantial fraction of the world’s population still lives in abject misery without even the benefit of adequate food and clean water?

At this time, molecular biologists have yet to join the ranks of the ultra‐rich. But there is no reason to suppose that being a molecular biologist somehow insulates us from the greed and megalomania that seems to infect those who become billionaires on the back of their inventions and investments. The most likely playgrounds in which a hypothetical molecular biologist might acquire such riches are in biotech and via the pharmaceutical industry. Big Pharma already enjoys low public esteem—in my view somewhat unfairly because its critics rarely take into account the real cost of R&D required to develop new drugs. But it is an easy target nonetheless, not least because of its scale, which inflates the bottom line of balance sheets in ways not easily justified in the eyes of “Joe Public”. Not to mention the many drugs that allegedly do not really work. In the public mind, we are already allied with corporate greed. It is not hard to see how this could become a reality.

Those who have accumulated extreme wealth in other sectors, and accrued power and influence to go with it, are viewed unfavorably by most people, even if some super‐rich individuals are admired for their creativity (Walker *et al*, 2021). Obscene wealth is still considered obscene, even if some of it is invested in scientific research, in the alleviation of human suffering, or in sponsorship of the arts. The fact that the ultra‐rich are able to do so seems to many like a usurpation of what is rightfully the responsibility of government, especially in democratic countries, and what do the wealthiest actually spend their money on anyway? Even if some of it is given away to charity, it is at the arbitrary whim of one or a few individuals, and not a transparent process with public accountability. Surely, it would be far better if they were all subject to a global wealth tax, the proceeds of which might support the UN, whose current annual budget (about US$ 3 billion) is mere pocket money for the world’s wealthiest persons.

But how could such a scheme actually work? Although the widespread sanctioning of those who supposedly bankroll the Kremlin seems to have had some effect, the ultra‐rich invariably devise ways of circumventing legal obstacles thrown in their path. There is a widespread suspicion that economic sanctions ultimately punish ordinary people rather than wayward decision‐makers or their wealthy friends. High taxes promote tax avoidance and tax havens. Strict regulations to prevent money laundering just fuel the rise of cryptocurrencies. Laws beget loopholes.

Global legislation with global implementation may look like an attractive tool, but its track record is not impressive. Consider the Kyoto/Paris process on climate change, for example, or the proposal for a 15% minimum corporate tax rate worldwide. Its effects are likely to be blunted by the floor effectively becoming the ceiling, so that most or all corporations could end up paying less and not more in taxes.

What about self‐regulation? Suppose EMBO made a rule that required any individual member to donate wealth accrued from molecular biology inventions above some threshold to charitable causes or be kicked out of the organization? Would such a person care? And if EMBO had any real power to frustrate their activities, would they not just be tempted to set up a rival organization where they would be free to continue their work without such restrictions?

To limit the wealth and power of individuals made rich by the capitalist market economy in which we all live, I think there is only one viable tool at our disposal, namely, the capitalist market economy itself. If we collectively boycott the products and services of companies owned by the very rich, unless they donate the bulk of their wealth to good causes of which we approve, we could eventually shift the balance in favour of better practices.

It may seem a pathetically inadequate tool and would certainly require a lot of time and effort. But in the long run, it could work. There is, for example, a growing movement to use market mechanisms to support ethical and environmentally sustainable businesses. Successful companies have adopted such advocacy in their own marketing, even if it sometimes seems a cynical ploy. They would not do so unless there is public demand. All we need is to add the accumulation of obscene wealth by company owners or majority shareholders as another abuse of the system that can potentially be reined in by consumer pressure. All it would take is for concerned individuals to devise a code of practice that could be used to judge compliance.

Oh well, maybe in the end, I should just take comfort in the fact that all the power of the ultra‐rich stems ultimately from mitochondria.

## Disclosure and competing interests statement

The author declares that he has no conflict of interest.
